# Supplementary material for: Current Advances in Humanized Mouse Models for Studying NK Cells and HIV Infection
Source: Microorganisms. 2023 Aug 2;11(8):1984. doi: 10.3390/microorganisms11081984 (PMC10458594; doi:10.3390/microorganisms11081984)
Supplement: Supplementary file 1 [file microorganisms-11-01984-s001.zip › microorganisms-2452694-supplementary.pdf]

Supplemental Table S1. Full name of mouse strains

**Abbreviated name**	**Mouse strains**
NSG	NOD.Cg-*Prkdc*^{scid} *Il2rg*^{tm1Wjl}/*SzJ*
NRG	NOD.Cg-*Rag1*^{tm1Mom} *Il2*^{rgtm1Wjl}/*SzJ*
NOG	NOD.Cg-*Prkdc*^{cid}*Il2rg*^{tm1Sug}/*JicTac*
BRG	C;129S4-*Rag2*$^{tm1.1Flv}$ *Il2rg*$^{tm1.1Flv}$/J
SRG	*SIRPA*$^{\mathrm{h/m}}$ *Rag2*$^{-/-}$ *Il2rg*$^{-/-}$
TKO	B6.129S-*Rag2*^{tm1Fwa}*CD47*^{tm1Fpl}*Il2r*$^{gtm1Wjl/J}$<br>C57BL/6.*Rag*$^{-/-}$*γc*$^{-/-}$*CD47*$^{-/-}$
NSG-Tg(IL-15)	NOD.Cg-*Prkdc*^{scid} *Il2rg*^{tm1Wjl} Tg(IL15)1Sz/SzJ
NOG-EXL	NOD.Cg *Prkdc*^{scid} *Il2rg*^{tm1Sug} Tg(SV40/HTLV-IL3,CSF2)10-7Jic/JicTac
NSG-SMG3	NOD.Cg-*Prkdc*^{scid} *Il2rg*^{tm1Wjl} Tg(CMV-IL3,CSF2,KITLG)1Eav/MloySzJ
MISTRG	C;129S4-*Rag2*$^{tm1.1FlvCsf1tm1(CSF1)Flv}$ *Csf2*/$^{Il3tm1.1(CSF2,IL3)Flv}$ *Thpo*$^{tm1.1(TPO)Flv}$ *Il2rg*$^{tm1.1Flv}$ Tg(SIRPA)1Flv/J
SRG-15	Balb/c x 129 *Rag2*$^{-/-}$ *Il2rg*$^{-/-}$ *hSIRPA KI hIL15 KI*
MISTRG-6-15	*Rag2*$^{-/-}$ *Il2rg*$^{-/-}$ MCSF$^{\mathrm{h/h}}$ IL3$^{\mathrm{h/h}}$ GMCSF$^{\mathrm{h/h}}$ *SIRPA*$^{\mathrm{h/m}}$ *THPO*$^{h/h}$ *IL6*$^{h/h}$ *IL15*$^{\mathrm{h/m}}$
NOG-IL15	NOD.Cg-*Prkdc*^{scid} *Il2rg*^{tm1Sug} Tg(CMV-IL2/IL15)1-1Jic/JicTac
hIL-7xhIL-15 KI NSG	NSG.CG-STOCK-IL7$^{\mathrm{tm1.1(Il7)Rk(IL15)Rk}}$
NSG-SMG3-IL15	NOD.Cg-*Prkdc*^{scid} *Il2rg*^{tm1Wjl} Tg(CMV-IL3,CSF2,KITLG)1Eav Tg(IL15)1Sz/J
